# Supplementary material for: Characteristics of Free Amino Acids (the Quality Chemical Components of Tea) under Spatial Heterogeneity of Different Nitrogen Forms in Tea (Camellia sinensis) Plants
Source: Molecules. 2019 Jan 24;24(3):415. doi: 10.3390/molecules24030415 (PMC6385162; doi:10.3390/molecules24030415)
Supplement: Supplementary file 1 [file molecules-24-00415-s001.pdf]

Supplementary Material:

Characteristics of Free Amino Acids (the Quality Chemical Components of Tea) under Spatial Heterogeneity of Different Nitrogen Forms in Tea (*Camellia sinensis*) Plants

Li Ruan[1, 2], Kang Wei[1], Liyuan Wang[1*], Hao Cheng[1*], Liyun Wu[1] and Hailin Li[1]

[1] National Center for Tea Improvement, Tea Research Institute, Chinese Academy of Agricultural Sciences, Hangzhou 310008, China; ruanli@tricaas.com; weikang@tricaas.com; wangly@tricaas.com; chenghao@tricaas.com; wuly@tricaas.com; lihailin@tricaas.com

[2] State Key Laboratory of Soil and Sustainable Agriculture, Institute of Soil Science, Chinese Academy of Sciences, Nanjing, 210008, China; ruanli@tricaas.com

* Correspondence: chenghao@tricaas.com; Tel.: +86-0571-86653169 (H.C.) wangly@tricaas.com; Tel.: +86-0571-86650575 (L.Y.W)

Figure S1. A chromatogram of the free amino acids (FAAs). Three graphs in each column represent three biological duplications in one treatment. The numbers on each peak represented the following FAAs: 1-Aspartic acid, 2-Serine, 3-Glutamic acid, 4-Glycine, 5-Histidine, 6-Arginine, 7-Threonine, 8-Alanine, 9-Proline, 10-Theanine, 11-Cysteine, 12-Tyrosine, 13-Valine, 14-Methionine, 15-Lysine, 16-Isoleucine, and 17-Leucine.

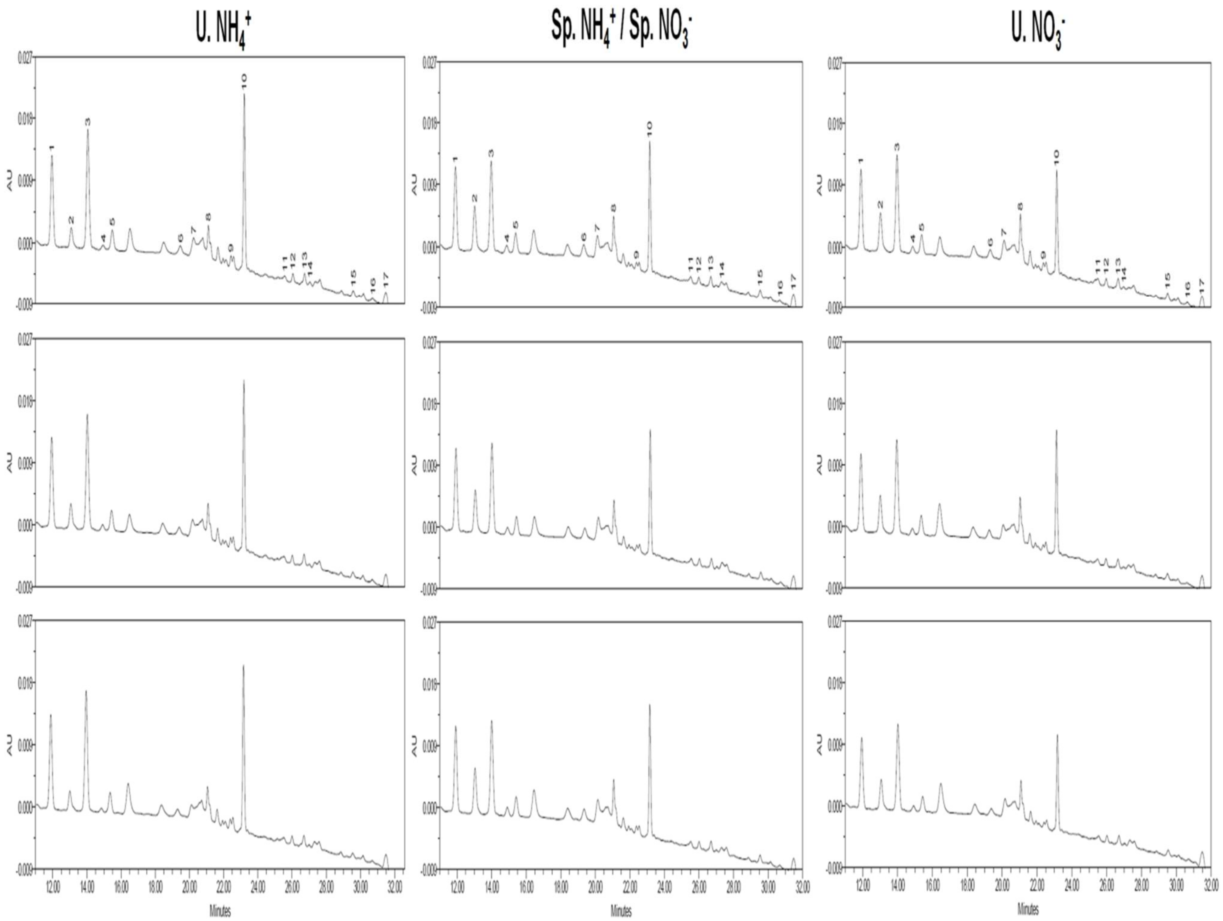

Table S1. The elution procedure in the experiment.

Time (min)	A%	B%	C%
0	100	0	0
0.5	99	1	0
18	95	5	0
19	91	9	0
29.5	83	17	0
33	0	60	40
36	100	0	0
45	100	0	0

Table S2. Quality control of the results.

Free amino acid	RSDs of retention time (%)	RSDs of peak area (%)
Aspartic Acid	0.203782	2.397479
Serine	0.183831	1.977028
Glutamic Acid	0.153178	1.375442
Glycine	0.153938	2.696006
Histidine	0.164164	4.155897
Arginine	0.168887	3.191873
Threonine	0.108757	0.78225
Alanine	0.059992	1.875245
Proline	0.012876	1.620989
Theanine	0.021654	3.333748
Tyrosine	0.065038	2.426868
Cysteine	0.056555	2.638419
Valine	0.069121	2.338318
Methionine	0.070099	2.210049
Lysine	0.067266	3.103045
Isoleucine	0.073026	2.444326
Leucine	0.074638	2.551925
